# Mucosal passive immunization with monoclonal antibodies targeting Candidalysin and Hyr proteins attenuates vaginal candidiasis in mice

**DOI:** 10.1080/21505594.2025.2569629

**Published:** 2025-10-09

**Authors:** Julie Auffray, Nicolas Biteau, Anne Cayrel, Denis Dacheux, Hassana Hsein, Pierre Tchoreloff, Thierry Noel

**Affiliations:** a University Bordeaux, CNRS, Microbiologie Fondamentale Et Pathogénicité, UMR 5234, Bordeaux, France; b University Bordeaux, CNRS, Bordeaux INP, Arts Et Metiers Institute of Technology, I2M Bordeaux, UMR 5295, Talence, France; c Bordeaux INP, Microbiologie Fondamentale Et Pathogénicité, UMR 5234, Bordeaux, France

**Keywords:** *Candida albicans*, vulvovaginal candidiasis, passive mucosal immunization, Candidalysin, Hyr1, monoclonal antibody

## Abstract

Vulvovaginal candidiasis (VVC) is a prevalent fungal infection primarily caused by the opportunistic pathogen *Candida albicans*. Among women affected by VVC, up to 8% experience more than four episodes per year, regarded as recurrent vulvovaginal candidiasis (RVVC). Current treatments for VVC are effective for isolated episodes but are insufficient for preventing recurrences, highlighting the need to develop novel treatments for the management of RVVC. In this study, we explore passive immunization as a potential alternative strategy to prevent VVC. The Candidalysin toxin (Cdlys) and the parietal protein Hyr1, specifically expressed by the infectious hyphal form of *C. albicans*, were selected as targets for murine mAbs development. Anti-Cdlys and anti-Hyr mAbs were produced using the hybridoma technology and were first selected for their antigenic specificity and high affinity. The anti-Hyr1 mAb 4F3.2.1 was then selected for its efficient opsonophagocytic activity with murine macrophages, while the anti-Cdlys mAb 5E2.2.1 was selected for its strong ability to neutralize the cytolytic and pro-inflammatory effects of Candidalysin. The prophylactic activity of the mAbs was then evaluated *in vivo* in a murine model of VVC. Results demonstrated that intravaginal administration of the combined mAbs confered protection against the development of the infection, significantly reducing fungal colonization and inflammation in the vaginal environment. These findings highlight the putative efficacy of passive mucosal immunization with anti-Cdlys and anti-Hyr1 mAbs in preventing VVC, providing a strong proof of concept for their potential as novel therapeutic strategy in the management of RVVC.

## Introduction

Vulvovaginal candidiasis (VVC) is an extremely common acute fungal infection. However, it is estimated that up to 8% of the affected women, i.e. several tens of millions of women worldwide, experience recurrent vulvovaginal candidiasis (RVVC), which is defined as more than four episodes occurring per year [1–3].

Most often, VVC is caused by the yeast *C. albicans* (90% of cases) [4]. *Candida albicans* lives as a commensal in humans, colonizing the oropharyngeal, gastrointestinal, and urogenital tracts. In response to different stimuli, *C. albicans* can switch from a yeast to a hyphal form, which contributes to its virulence during mucosal infections. This morphological transition is associated with the expression of several specific proteins that facilitate adhesion and invasion of the hyphae to host cells, such as Hwp1 (Hyphal wall protein 1) and Als (Agglutinin-like sequence) proteins. Additionally, *C. albicans* hyphae secrete proteases like Saps (Secreted aspartic proteinases) that contribute to tissue damage [5,6]. The hyphal form of *C. albicans* is also associated with the expression of Ece1 (Extent of Cell Elongation) [7], which, after maturation through endoproteolysis, generates a potent cytolytic peptide toxin of 31 amino acids named Candidalysin [8] (Cdlys). In the context of VVC, this toxin has been identified as a major contributor to cell damage and as a key effector of the inflammatory response by inducing the secretion of pro-inflammatory cytokines, which are involved in recruiting macrophages and polymorphonuclear neutrophils (PMNs) to the vaginal mucosa [9].

Recent advances suggest that VVC could result from a dysfunction of local innate immunity, due to PMN anergy [10]. The inability of PMNs to clear *C. albicans*, combined with the pro-inflammatory effect of the Cdlys toxin, is thought to contribute to the installation of an inflammatory loop that intensifies the severity of the symptoms during infection [10,11].

Current treatments for acute VVC rely on the use of fungistatic azole antifungals. Although they are very effective, these treatments fail to provide protection against recurrences [12,13]. Furthermore, repeated use of azole-based therapy carries the risk of exerting selection pressure on the emergence of *Candida* strains less susceptible to azole treatments [14,15]. These considerations led us to explore alternative therapeutic strategies for RVVC treatment that could complement or even replace the current arsenal of conventional therapies.

Immunotherapy based on passive immunization relies on the administration of antibodies for the treatment or prevention of a disease. This approach has long been used to neutralize microbial toxins [16,17] and has since been extended to the therapeutic management of various pathologies, including infectious diseases [18], autoimmune disorders, and inflammatory diseases such as Crohn’s disease and rheumatoid arthritis [19–21]. Considering that (R)VVC can result from immunopathological disorders, we hypothesized that passive immunoprophylaxis could represent an alternative solution to counteract the uncontrolled inflammation mediated by Candidalysin and by neutrophils anergy during (R)VVC infections.

Among the various hypothetical molecular mechanisms that could explain neutrophil anergy, the Hyr1 cell surface protein specifically expressed by the hyphal form of *C. albicans* has been reported to be possibly responsible for masking immunogenic antigens, thus falicitating its escape from phagocytosis [22]. Furthermore, Hyr1 was already described as an anti-infectious immunotherapeutic target [23] and successfully used in preclinical vaccine trials against *C. albicans* [24]. In the present article, we first selected and characterized mAbs in mice raised against two targets specifically expressed by the hyphal form of *C. albicans*: the Cdlys toxin and the cell wall protein Hyr1. We then conducted a proof-of-concept study to evaluate the efficacy of passive immunization using anti-Cdlys and anti-Hyr1 mAbs in a mouse model of VVC, anticipating distinct but complementary biological actions for each antibody, i.e. neutralizing the cytolytic and pro-inflammatory effects of Cdlys and opsonizing the hyphae of *C. albicans* to enhance the phagocytic potential of immune cells. Our results showed that the combined intravaginal administration of anti-Hyr1 and anti-Cdlys mAbs efficiently reduces the inflammatory response in the vaginal environment and decreases the fungal burden in the vaginal mucosa in the context of prophylactic treatment for VVC.

## Materials and methods

### Ethics statement

Mice experiments have been performed in the conventional animal facilities of the University of Bordeaux (France) (approval number of B33036917 and C33063916), under the approval of institutional guidelines determined by the local Ethical Committee of the University of Bordeaux and under approval of the French Ministry for Higher Education and Research and the French Committee of Genetic Engineering : approval number APAFIS (Autorisation de projet utilisant des animaux à des fins scientifiques) #17621- 2,018,112,201,234,223 v5 for mice immunization and antibodies production, and APAFIS #33116-2021091709119827 v3 for vulvo-vaginal candidiasis experiments in mice. We also adhere to the ARRIVE ghuidelines and provide a check list that can be found at DOI: https://doi.org/10.17632/3r9dghk85h.2.

### Fungal strains and culture conditions

*C. albicans* strains (Table 1) were cultured in YPD medium (yeast extract (Difco) 1% (m/v), Bacto^TM^peptone (Difco) 2% (m/v), D-glucose (Euromedex) 2% (m/v), with agar (Euromedex) 2% (m/v) for solid medium) at 30°C, shaken at 220 rpm for liquid cultures. Hyphal growth was induced in RPMI 1640 (Gibco) supplemented with Fetal Bovine Serum (FBS, Eurobio Scientific) 10% (v/v) at 37°C under 5% CO_2_. Cells from overnight cultures were washed twice with phosphate-buffered saline (PBS) and adjusted to the assay-required cell concentration in the respective assay media.

**Table 1.** *Candida albicans* strains used in this study.

Strain	Genotype	Description	Reference
SC5314	Wild-type	ATCC	[25]
BWP17	*ura3*∆/∆, *arg4*∆/∆, *his1*∆/∆	Uridine, arginine, and histidine auxotrophic strain derived from SC5314	[26]
*ece1*∆/∆	*his1*∆/∆, *ece1*∆::*URA3*/*ece1*∆::*ARG4*	*ECE1* replaced by *URA3* and *ARG4* cassettes in BWP17, auxotrophic for histidine	This study
*hyr1*∆/∆	*his1*∆/∆, *hyr1*∆::*URA3*/*hyr1*∆::*ARG4*	*HYR1* replaced by *URA3* and *ARG4* cassettes in BWP17, auxotrophic for histidine	This study
CAI-4	*ura3*∆/∆	Uridine auxotrophic strain derived from SC5314	[25]
CAI-4-gLUC	*ura3*∆, *RPS1::CIp10-Act1p-PGA59-gLUC-URA3*	Constitutive gLUC expression in CAI-4 strain via CIp10:ACT1p-gLUC59-*URA3* integration at the *RPS1* locus	This study

### Mammalian cell culture

The A431 vaginal squamous cell carcinoma cell line (ECACC 85,090,402) was cultured at 37°C and under 5% CO_2_ in Dulbecco’s Modified Eagle Medium (DMEM) with 4.5 g/L glucose (Gibco®), supplemented with FBS 10% (v/v) and antibiotics (penicillin/streptomycin, 50 μg/mL). To ensure optimal growth, the medium was refreshed every two days, facilitating nutrient replenishment and removal of dead cells. For cell harvesting, the adherent cell monolayer was rinsed with 10 mL of PBS then treated with 10 mL of trypsin-EDTA (Eurobio Scientific) and incubated for 15 min at 37°C to facilitate detachment. Following detachment, cell counting was carried out using a Malassez hemocytometer, and the cells were resuspended at the desired concentration for further experiments.

The macrophages derived from the murine cell line J774A.1 (ECACC 91,051,511), used for phagocytosis experiments, were cultured under the same conditions as those described for the A431 cell line. However, for infection assays, the cells were harvested using a sterile cell scraper (Corning Inc.), without the use of trypsin.

### Peptides

The peptide sequences SIIGIIMGILGNIPQVIQIIMSIVKAFKGNK, corresponding to Candidalysin (KEGG accession number CAALFM_C403470CA), and LQLRADALPQYFKIGKGYDSKLFRIVNSRGLKN, corresponding to the N-terminal part (aa 274–322) of Hyr1 (KEGG accession number CAALFM_C113450WA), along with their corresponding keyhole limpet hemocyanin (KLH)-conjugated versions, were synthesized and purified by Covalab R&D in Biotechnology (Bron, France).

### Hybridoma technology for the production of anti-Hyr1 and anti-cdlys mAbs

Balb/c mice (9 weeks old, Charles Rivers Laboratories) were used for antibody production. Pre-immune serum was collected for testing five days before immunization using indirect ELISA. On the day of immunization, 10–20 μg of the antigenic peptide was injected into the hind footpads of the mice using complete Freund’s adjuvant (Sigma). After twelve days, a booster immunization was administered with incomplete Freund’s adjuvant (Sigma). Serum samples were collected three days later and tested by indirect ELISA. Mice exhibiting positive serum tests were euthanized, and popliteal lymph nodes were harvested.

To extract B lymphocytes, popliteal lymph nodes were crushed in a petri dish with RPMI 1640 medium at 37°C. The suspension was transferred to a 15 mL tube and left to settle at 37°C for a few minutes. The cells were then collected, centrifuged (1400 rpm, 5 min), and washed twice with RPMI 1640. After counting, an equal number of B lymphocytes from popliteal lymph nodes and P3U1 myeloma cells were separately centrifuged, and the cell pellets were resuspended in RPMI 1640. The two suspensions were mixed and centrifuged (1400 rpm, 5 min), and the supernatant was discarded. The pellet containing myeloma cells and B lymphocytes was incubated at 37°C, and 1 mL of polyethylene glycol (PEG 1500) (Roche Diagnostics) was gradually added. The suspension was transferred into a flask, and 144 mL of complete HAT RPMI medium (RPMI 1640 supplemented with heat-inactivated FBS 8% (v/v), β-mercaptoethanol (0.05 mM), sodium pyruvate (2 mM), non-essential amino acids (1X) (Gibco), HAT (1X) (Hybri-MaxTM, Sigma), and antibiotics (penicillin/streptomycin, 50 μg/mL)) was added. The cell suspension was distributed into 96-well plates (200 μL/well), which were then incubated at 37°C and under 5% CO_2_. Seven days later, 100 μL of medium was replaced with fresh complete HAT RPMI medium. Hybridoma cells typically emerged around 15 days after fusion. The supernatant from the hybridomas was tested by indirect ELISA, and positive hybridomas were cloned on the same day using the limiting dilution method in complete HAT RPMI medium

### Indirect enzyme-linked immunosorbent assay (ELISA)

Nunc Maxisorp 96-well plates (Thermo Fisher Scientific) were coated with the antigen (1 μg/well, diluted in bicarbonate buffer (50 mM, pH 9.6)) and incubated overnight at 4°C. The following day, the wells were blocked with 200 μL of a PBS/gelatin (0.2% (w/v)) solution for 30 min at 37°C. After removing the blocking buffer, the plates were washed three times with 300 μL of a PBS/Tween-20 (0.05% (v/v)) solution and 100 μL of supernatant was added to each well. The plates were then incubated for 1h30 at 37°C. After incubation, the plates were washed again and incubated for 1 h at 37°C with 100 μL/well of horseradish peroxidase-conjugated sheep anti-mouse IgG (H+L) (Jackson ImmunoResearch, #515–035–062), diluted 1:5000 in PBS. Following another washing step, 100 μL/well of a solution of citric buffer (50 mM, pH 4) containing H_2_O_2_ (0.03% v/v) and ABTS substrate (2,2’-azino-bis(3-ethylbenz-thiazoline-6-sulfonic acid)) (40 μM) was added. The plates were incubated for 20 min at room temperature and the absorbance was measured at 405 nm using a Fluostar Omega plate reader (BMG Labtech).

### MAbs purification and isotype determination

Secreted antibodies were purified of culture supernatant by protein G column “HiTrapTM Protein G HP 1 mL” using AKTA pure^TM^ system (GE Healthcare). Before purification, hybridoma supernatants were filtered (0.45 µm). A sodium phosphate buffer (20 mM, pH 7) was used for column equilibration and washing. The elution was performed in 500 μL fractions using glycine buffer (100 mM, pH 2.7), and the eluates were collected in pre-filled tubes containing 100 μL of Tris buffer (1 M, pH 9) for immediate buffering. Eluates were analyzed on SDS-PAGE gel stained with Coomassie Blue to control the efficiency of the purification. Purified samples were stored at 4°C with sodium azide 0.02% (v/v).

The mAbs isotypes were identified using the IsoStripTM Mouse Monoclonal Antibody Isotyping kit (Santa Cruz, #SC-24958) in accordance with the supplier’s recommendations.

### Immunofluorescence assays

#### Immuno-detection of Hyr1

*Candida albicans* cells were seeded onto 12 mm glass cover slips at a density of 1 × 10^6^ cells/well in RPMI 1640 containing FBS 10% (v/v) and incubated for 2 h at 37°C and under 5% CO_2_. Unbound cells were removed by washing with PBS, and adhered cells were fixed in 4% paraformaldehyde (PFA) for 30 min at room temperature. After three washes in PBS, fungal cells were treated with blocking buffer (PBS, Tween-20 0.01% (v/v), BSA 1% (w/v)) for 20 min at room temperature and immunostained by adding 100 μL of anti-Hyr1 hybridoma culture supernatant for 1 h at room temperature. Samples were washed twice with blocking buffer and incubated with a fluorescent secondary antibody (Sigma, #F2012) (1:5000 in blocking buffer, 1 h, in the dark). The glass cover slips were mounted with Slowfade® Gold antifade reagent (Molecular Probes) and visualized by fluorescence microscopy.

#### Binding kinetic study of anti-Hyr1 4F3.2.1 mAb

*Candida albicans* cells were seeded in a six-well plate at a density of 1 × 10^6^ cells/well in RPMI 1640 containing 10% (v/v) FBS or YNB (Yeast Nitrogen Base) medium and incubated for various durations at 37°C. As described above, the samples were washed with PBS and fixed with 4% paraformaldehyde (PFA). The fungal cells were then harvested using a sterile scraper, centrifuged (2000 *g*, 5 min), and washed twice before being resuspended in PBS.

Twenty microliters of the cell suspension was added to each well of a multi-well slide previously coated with a polylysine solution (Sigma) (0.1% w/v in sterile water) and incubated for 15 min at room temperature. After three washes in PBS, samples were incubated with blocking buffer (PBS, Tween-20 0.01% (v/v), BSA 1% (w/v)) for 20 min at room temperature. Samples were immunostained with anti-Hyr1 4F3.2.1 mAb (5 µg/mL in blocking buffer, 1 h, in the dark) and a fluorescent secondary antibody (Sigma, #F1220) (1:5000 in blocking buffer, 1 h, in the dark). The samples were washed and the nuclei were stained for 5 min using a solution of 10 µg/mL DAPI (4,’6-diamidino-2-phenylindole). The slides were mounted with Slowfade® Gold antifade reagent (Molecular Probes) and visualized by fluorescence microscopy.

#### Immuno-detection of Candidalysin

A431 cells were plated on 12 mm glass cover slips at 5 × 10^5^ cells/well in RPMI 1640 containing FBS 10% (v/v) and incubated for 48 h at 37°C under 5% CO_2_. On the day on which the experiment was to be conducted, unbound cells were removed by washing with RPMI 1640 (without FBS), and *C. albicans* strains added at 2.5x10^3^ cells/well and incubated for 1.5 hour at 37°C and under 5% CO_2_. A431 plasma membrane was labeled for 10 min at room temperature by adding a solution of 0.2X of dye Cell Mask Deep Red (Thermoscientific). Cells were washed three times in PBS and samples were fixed with 4% paraformaldehyde (PFA) for 15 min at room temperature. After three washes in PBS, external fungal hyphae were stained for 30 min at room temperature using a solution of 5 μg/mL fluorescent conjugated Concanavalin A (Invitrogen, #C21421). Samples were washed in PBS three times and treated with blocking buffer (PBS, Tween-20 0.01% (v/v), BSA 1% (w/v)) for 30 min at room temperature. Samples were immunostained with anti-Cdlys 5E2.2.1 mAb (1:100 in blocking buffer, 1 h, in the dark) and a fluorescent secondary antibody (Sigma, #F1220) (1:5000 in blocking buffer, 1 h, in the dark). After three washes, cell nuclei were stained adding 10 µg/mL of DAPI solution. The glass cover slips were mounted with Slowfade® Gold antifade reagent (Molecular Probes) and visualized by fluorescence microscopy.

### Hemolysis assay

Anti-Cdlys mAbs were assessed for their ability to neutralize Candidalysin in a hemolysis assay. For each antibody tested, 10 µL of sheep red blood cells (RBCs) (20 µL of 50% sheep RBCs in Alsever buffer, Boehringer Ingelheim Therapeutics GmbH) were utilized. The RBCs/Alsever’s suspensions were centrifuged in plastic test tubes (800 *g*, 5 min), and the supernatant was carefully removed. The RBCs were washed by gently suspending them in 1 mL of PBS and centrifuged as described above. The supernatants of each hybridoma cell line were supplemented with Candidalysin (20 μM) and incubated for 2 h at 37°C. The remaining RBC pellets were gently suspended in 100 µL of the supernatants mixed with Candidalysin and incubated for 6 h at 37°C with gentle inversion of the tubes every 30 min to maintain RBCs in suspension. After incubation, the samples were centrifuged (800 *g*, 5 min), and 70 μL of the supernatants were transferred to a fresh 96-well plate. Finally, absorption was measured at 540 nm using a Fluostar Omega plate reader (BMG Labtech). Hemolysis was defined as the absorbance of the samples relative to the absorbance of a reference lysis sample (RBCs incubated with Candidalysin in a non-relevant hybridoma cell line supernatant) following the subtraction of the absorbance from the negative control (RBCs incubated with supernatants, without Candidalysin).

### Surface plasmon resonance (SPR)

Surface plasmon resonance was used to determine the binding affinity of mAbs. Experiments were conducted at 25°C using a Biacore T200 system (GE Healthcare Life Science) with a pre-filtered and degassed PBS/Tween 20 running buffer (0.05% (v/v)). Peptide ligands were prepared at concentrations of 10–20 μg/mL in sodium acetate buffer at pH 5–7. These ligand solutions were injected onto the surface of a flow cell in a CM5 chip (GE Healthcare Life Science) and ligands were immobilized by amine coupling, following the manufacturer’s recommendations. One track on the chip was left blank for double referencing of the sensorgrams. Antibodies were dialyzed against the running buffer and injected onto their respective targets using a Single Cycle Kinetic (SCK) approach. This method involved sequential injection at increasing concentrations without chip regeneration between injections. Antibodies were injected for 60 seconds at three increasing concentrations (50, 100, 200 mM) with a dissociation time of 240 seconds. After each SCK cycle, the chip was regenerated for 30 seconds with 10 mM NaOH solution. Sensorgrams were analyzed using Biacore T200 2.0 evaluation software (BiacoreTM). The association (ka) and dissociation (kd) rate constants were determined by directly fitting the sensorgram curves to a 1:1 Langmuir interaction model. The equilibrium dissociation constant, KD, was calculated as kd/ka.

### Phagocytosis assay

J774 macrophages were plated in a culture-treated white 96-well plates with clear well bottoms (Greiner Bio-one) (2x10^5^ cells/well) in DMEM containing 10% (v/v) FBS and incubated overnight at 37°C under 5% CO_2_. Yeast cells were collected from overnight culture in YPD supplemented with 5 µg/ml Calcofluor White (CFW, Sigma) and adjusted to the required concentration in complete RPMI (cRPMI: RPMI-1640 (Sigma) without phenol red and supplemented with FBS (10%), 1 mM sodium pyruvate and 2 g/L sodium bicarbonate) plus 5 µg/ml CFW. The cell suspension was supplemented with the anti-Hyr1 4F3.2.1 mAb at the required concentration depending on the mAb/yeast ratio tested. Depending on the experiment, the mixture was then incubated for 30 min at 37°C to induce filamentation. J774 macrophages were infected with 200 µl of opsonized CFW-labeled yeasts or hyphae (sixtuplets of wells were prepared for each condition) and incubated for 1 h at 37°C and under 5% CO_2_. As a control, uninfected phagocytes were stained with 5 µg/µl CFW in cRPMI. PBS alone was used as a negative fluorescent control.

Following incubation, the plate was centrifuged (2200 *g*, 5 min), and the wells were washed with PBS. For the quenching experiment, 200 µL of PBS was added to three wells of each condition to determine the total fluorescence of CFW-labeled yeasts (both intra-macrophagic and extracellular), while the remaining three wells were treated with 200 µL of trypan blue (1 mg/mL) specifically to detect the fluorescence from internalized CFW-labeled yeasts. After washing the wells with PBS, 200 µL of PBS was added, and the CFW fluorescence (λex 350 nm, λem 460 nm) was measured using a FluoStar Optima fluorimeter (BMG Labtech). After subtracting the residual fluorescence not attributable to yeasts (uninfected macrophage condition), the fluorescence value after quenching was compared to the total fluorescence in PBS in order to determine the percentage of internalized yeasts.

### Epithelial cell damage assay

The neutralizing activity of the anti-Cdlys mAb 5E2.2.1 was assessed by examining its capacity to reduce damage generated in A431 epithelial cells treated with Candidalysin. In this experiment, 50 μL of A431 cell suspension at a density of 2 × 10^5^ cells/mL in DMEM with 10% FBS was plated in a 96-well plate and incubated for 2 h at 37°C under 5% CO_2_. Adhered cells were then treated with the synthetic Candidalysin peptide (5 μM), either alone or in combination with the mAb 5E2.2.1 (1.25 μM), or a non-relevant mAb (negative control), and incubated for 24 h at 37°C under 5% CO_2_. After incubation, the cellular damage was assessed by quantifying the enzymatic activity of lactate dehydrogenase (LDH) in the culture supernatant using a CytoTox 96 nonradioactive cytotoxicity assay (Promega) following the manufacturer’s instructions. LDH from porcine heart (Sigma) was used to create a standard curve ranging from 2 to 40 units of enzymatic activity/mL.

### Cytokine release

Much like in the cell damage assay, human cytokines IL-1α and IL-8 were measured in the culture supernatant of A431 cells treated with Candidalysin alone or in combination with the mAb 5E2.2.1 (2.5 μM) to assess whether mAb 5E2.2.1 can inhibit the toxin’s pro-inflammatory activity. The protocol used here was similar to the one described earlier, with the exception that the infection was conducted on 5 × 10^4^ A431 cells. Furthermore, the Candidalysin concentration for infection was set at 5 and 10 μM for the quantification of the cytokines IL-8 and IL-1α, respectively. The cytokine levels were determined using Mini ABTS ELISA Development kits (Peprotech, #900-M11, #900-M18) following the manufacturer’s recommendations.

### Generation of the bioluminescent strain of *Candida albicans* CAI-4-gLUC and of the *ece1*∆/∆ and *hyr1*∆/∆ mutant strains

The CIp10:Act1p-gLUC59 plasmid was used in this study [27]. It contains the full gene of *Gaussia princeps* gLUC luciferase, whose expression is driven by the constitutive *Candida* promoter *ACT1*. The gLUC gene is linked to the C-terminal part of the GPI anchor of the PGA59 parietal protein of *C. albicans*, for allowing the expression of luciferase at the surface of fungal cells. The *C. albicans* bioluminescent strain CAI-4-gLUC was obtained through transformation of the CAI-4 strain using StuI-linearized CIp10:Act1p-gLUC59. The proper integration into the *RPS1* locus of *C. albicans* was confirmed by PCR.

Bioluminescence of transformed yeast cells and controls was measured in cells collected from overnight culture in YPD, washed with PBS and adjusted to the required concentration in Dulbecco’s phosphate-buffered saline (DPBS) (NaCl 137 mM, KCl 2.7 mM, Na_2_HPO_4_ 10 mM, KH_2_PO_4_ 1.76 mM, pH 7.4), supplemented with 2 μM of coelenterazine (Invitrogen). The cell suspensions were plated in a 96-well microplate and incubated for 15 min in the dark. Luminescence intensity was acquired using a FluoStar Optima fluorimeter (BMG Labtech).

The growth of the strains was tested using the drop test method. Drops of 5 μL of a suspension of SC5314, CAI-4 and CAI-4-gLUC strains, ranging from 1 × 10^7^ to 1 × 10^3^ cells/mL, were spotted onto various solid media. The plates were incubated at 35°C for 48 h and photographed using the ImageQuantTM LAS 4000.

The *ece1*∆/∆ and *hyr1*∆/∆ mutant strains were generated from the BWP17 *Candida albicans* strain by the deletion of each *ECE1* or *HYR1* alleles (GeneBank accession numbers XM_706502 and XM_Z50123, respectively), using a methodology already described [28]. Deletion cassettes containing the selectable markers *URA3* or *ARG4* from the *C. albicans* wild type strain SC5314 (GenBank accession numbers XM_716694 and XM_707049, respectively) fused to the 5’UTR and 3’ UTR regions of the target gene *ECE1* or *HYR1* were constructed using the In-Fusion HD Cloning Kit (Clontech, USA). Deletion cassettes were transformed to BWP17 yeast cells by electroporation, and the precise locus-specific integration of each deletion cassette via homologous recombination was verified by PCR and nucleotide sequencing.

### Murine model of vulvovaginal candidiasis and mAb prophylaxis treatment

Upon arrival, BALB/cBy female mice (6 weeks old, Charles River Laboratories) were housed in groups of five mice per cage and underwent a one-week acclimatization period before starting experiments.

To induce pseudo-estrus, mice were administered with 0.2 mg of β-estradiol 17-valerate (Sigma), dissolved in 100 μl of sesame oil (Sigma), via subcutaneous injection five days prior to inoculation (day −5) as well as on day two.

Two days before the first day of treatment (day −7), purified eluates of anti-Cdlys 5E2.2.1 and anti-Hyr1 4F3.2.1 mAbs were dialyzed against PBS using a Spectra/Por® porous membrane (Spectrum Laboratories) and stored at 4°C. Starting on day −5 and continuing for six consecutive days, anesthetized mice (isoflurane 5%; 1 l/min air and 1 l/min oxygen) were intravaginally treated with 20 μl of a mixture of the two mAbs (prepared at a final concentration of 1, 10 or 50 μg/20 μl per mAb). Untreated animals received only PBS.

On day 0, anesthetized mice were intravaginally infected with 1 × 10^7^ *C. albicans* CAI-4-gLUC yeast cells suspended in 20 μL of PBS. The development of the infection was monitored every 48 to 72 h post-infection. For each imaging session, the lower ventral part of the anesthetized animals was shaved, and 20 μL of coelenterazine (0.5 mg/mL in 1:8 EtOH:PBS, v/v) was intravaginally injected into the mice. The animals were then imaged using the PhotonIMAGER RT (Biospace) for 20 min. The total emission of photon flux detected in the vaginal region was quantified using the same Region Of Interest (ROI) for all animals and procedures.

At the end of the experiment, the animals were euthanized by cervical dislocation. Immediately after euthanization, a vaginal lavage was performed (3 ×50 μL PBS) to assay cytokines. The levels of the pro-inflammatory immunomodulators IL-1α and MIP-2 in the vaginal samples were determined using Mini ABTS ELISA Development kits (Peprotech, #900-K152, #900-K82) according to the manufacturer’s recommendations.

## Results

### Production and selection of anti-Hyr1 and anti-cdlys mAbs

#### Production of anti-Hyr1 mAbs

Hyr1 is a 919 amino acid GPI-anchored cell wall protein of *C. albicans* specifically expressed at the hyphal stage [29]. The surface-exposed N-terminal part of the protein was specifically targeted for generating antibodies directed against Hyr1. The sequence of the selected peptide corresponds to amino acids 274 to 322 of Hyr1 (LQLRADALPQYFKIGKGYDSKLFRIVNSRGLKNAVTYDGPVPNNEIPAV); this peptide sequence overlaps with the sequence of two peptides already shown to be effective for the production of polyclonal antibodies directed against Hyr1 in rabbits [24].

**Figure 1.**
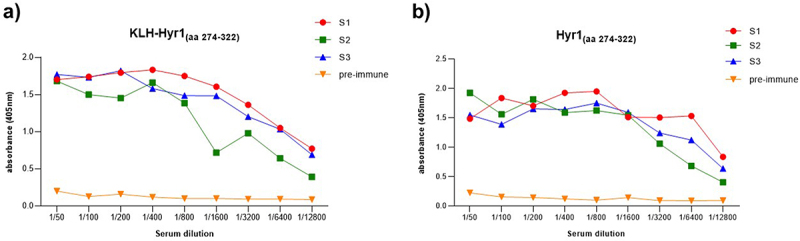
ELISA titration of mouse antiserum after immunization with KLH-Hyr1_(aa 274–322)_. (a) Plates coated with KLH-Hyr1_(aa 274–322)_. (b) Plates coated with Hyr1_(aa274-322)_. Serum samples from three immunized mice (S1, S2, and S3) were analyzed. Pre-immune serum from mouse S1 (collected prior to immunization) was included as a negative control.

The KLH-conjugated peptide Hyr1_(aa 274–322)_ was used to immunize mice to produce Hyr1-targeting antibodies. Three days after the booster injection, antiserums were collected from animals and screened for Hyr1 reactivity and specificity by ELISA assay. All antiserums were found to contain antibodies specifically directed against KLH-Hyr1_(aa 274–322)_ and against Hyr1_(aa 274–322)_ confirming the activation of a humoral response, with a comparable intensity in each individual animal (Figure 1).

Following this, splenocytes were isolated from mice and fused with myeloma cells for hybridoma production. After screening using the limiting dilution method, eight monoclonal hybridoma populations were selected by ELISA based on the secreted antibodies’ high capacity to bind to Hyr1_(aa 274–322)_ or to KLH- Hyr1_(aa 274–322)_, and their low cross-reactivity with structurally unrelated antigens such as BSA and tubulin (Table 2).

**Table 2.** Representative results from hybridoma screening performed using indirect ELISA.

	Hybridoma cell line							
Antigen	12D4.1.1	12E11.1.1	12D5.1.1	12G8.1.1	4F3.2.1	4D6.2.1	4G3.1.1	12C10.4.2
Hyr1_(aa 274–322)_	1.907	1.778	1.152	1.519	1.49	1.802	1.937	2.033
BSA	0.222	0.191	0.103	0.073	0.084	0.097	0.137	0.115
Tubulin	0.179	0.079	0.14	0.073	0.091	0.084	0.115	0.105
KLH-Hyr1_(aa 274–322)_	2.005	1.864	1.405	1.5	1.503	1.702	1.835	1.858

The data represent OD_405 nm_ values measured from eight selected hybridoma cell line supernatants obtained after immunizing mice with KLH-Hyr1_(aa 274–322)_. The binding specificity of antibodies was assessed by comparing their binding to plate wells coated with Hyr1_(aa 274–322)_ or KLH-Hyr1_(aa 274–322)_ peptides, and with unrelated antigens such as BSA and tubulin.

#### Binding of anti-Hyr1 mAbs to native Hyr1

**Figure 2.**
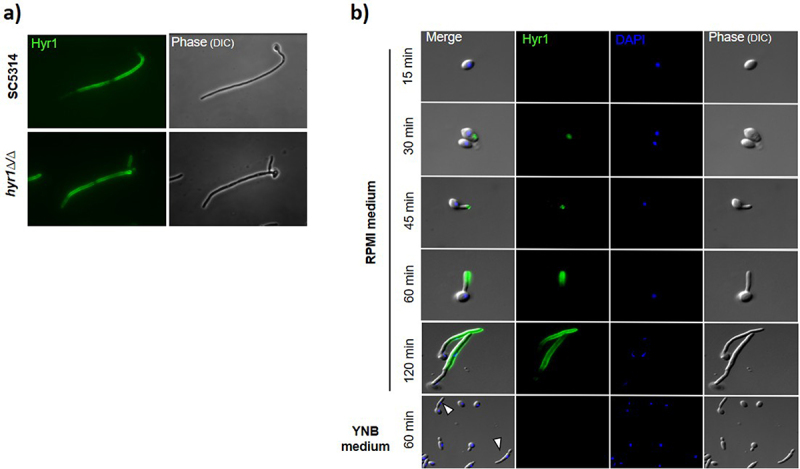
Immunofluorescence imaging of anti-Hyr1 4F3.2.1 mAb. (a) The binding specificity of anti-Hyr1 4F3.2.1 mAb to the hyphal cell wall protein Hyr1 of *C. albicans* was investigated by immunofluorescence assays. *C. albicans* SC5314 (WT) and *hyr1∆/∆* mutant yeast strains were cultivated for 2 h in RPMI with 10% FBS to induce filamentation and then incubated with culture supernatant from the hybridoma cell line 4F3.2.1. Murine antibodies were detected using fluorescently labeled goat anti-mouse antibodies. (b) immunofluorescence analysis of the binding kinetics of anti-Hyr1 4F3.2.1 mAb on *C. albicans* hyphal cells. SC5314 yeast cells were cultivated in RPMI medium with 10% FBS to induce filamentation or in YNB medium to promote pseudo-hyphal formation (white arrows). Cells at various stages of elongation were co-stained with purified anti-Hyr1 4F3.2.1 (detected with a green fluorescent secondary antibody) and DAPI nuclear staining dye (blue).

The ability of the selected anti-Hyr1_(aa 274–322)_ mAbs to recognize the native form of the protein Hyr1 expressed at the fungal cell wall was investigated using indirect immunofluorescence assay. Only one of the eight secreted mAbs, 4F3.2.1, was found to bind wild type (WT) *C. albicans* hyphae. However, cross-binding to the hyphae of a *hyr1*∆/∆ mutant strain used as a control showed that the anti-Hyr1 4F3.2.1 also recognized cell wall proteins of *C. albicans* differing from Hyr1 (Figure 2(a)).

We then studied the binding kinetics of anti-Hyr1 4F3.2.1. Yeasts cells were grown under conditions promoting filamentation (RPMI/FBS 10%, 37°C) before being immunolabeled at various stages of elongation with the selected mAb (15, 30, 45, 60, and 120 minutes). Yeasts were also cultivated in YNB medium, known to promote their growth under yeast and pseudohyphal forms. Immunofluorescence analysis indicated that anti-Hyr1 4F3.2.1 bound to the hyphae very early during differentiation (30 min) and bound the entire hyphae during elongation (120 min). Additionally, the antibody was not witnessed binding to other morphological forms -yeast and pseudohyphae- demonstrating that the antibody was specific to the hyphal form (Figure 2(b)).

Consequently, the previously observed polyspecificity of anti-Hyr1 4F3.2.1 for other cell wall proteins is limited to the hyphal stage. This characteristic was seen as an advantage for its expected biological properties, as increasing the number of potential mAb binding sites on hyphae is likely to enhance the efficiency of opsonization.

#### Production of anti-cdlys mAbs

**Figure 3.**
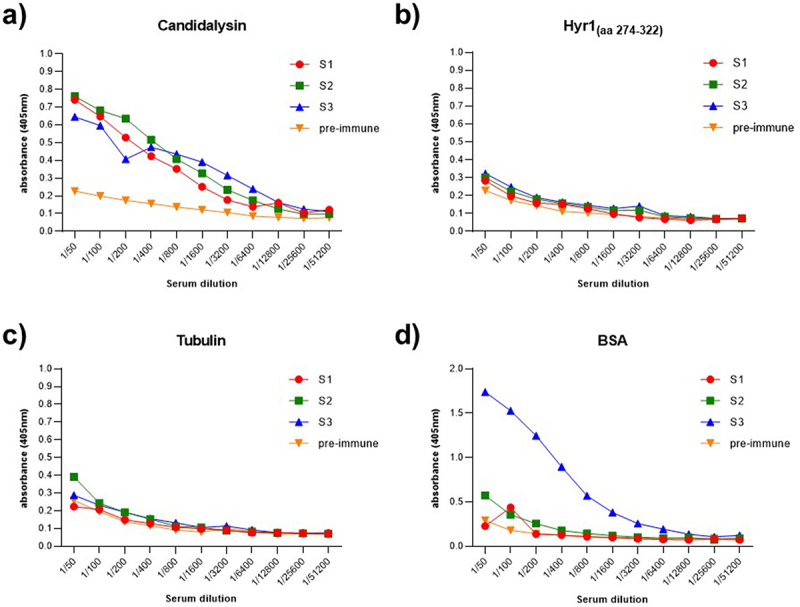
ELISA titration of mouse antiserum after immunization with Candidalysin. (a) Plates coated with Candidalysin, and unrelated antigens (b) Hyr1_(aa274-322)_, (c) tubulin, and (d) BSA. Serum samples from three immunized mice (S1, S2, and S3) were analyzed. Pre-immune serum from mouse S1 (collected prior to immunization) was included as a negative control.

The full-length 31 amino acid Candidalysin peptide (SIIGIIMGILGNIPQVIQIIMSIVKAFKGNK) was used to immunize three mice to produce Cdlys-targeting antibodies. As previously described, antiserum reactivity for the antigen was evaluated three days following the administration of the booster injection using indirect ELISA. To assess the specificity of the humoral response, the reactivity of each antiserum was also tested against unrelated antigens, including BSA, tubulin, and Hyr1_(aa 274–322)_. Compared to the pre-immune control serum, the antiserum from immunized mice demonstrated a significant increase in antigenic reactivity when tested against the specific Cdlys (Figure 3). No cross-reactivity was detected in S1 and S2 antiserums, whereas S3 demonstrated high reactivity against BSA. As a result, plasmocytes were harvested for hybridoma production only from immunized mice 1 and 2. Ten monoclonal hybridoma populations secreting specific anti-Cdlys mAbs were selected following limiting dilution screening method.

#### Study of the neutralizing activity of anti-cdlys mAbs

**Figure 4.**
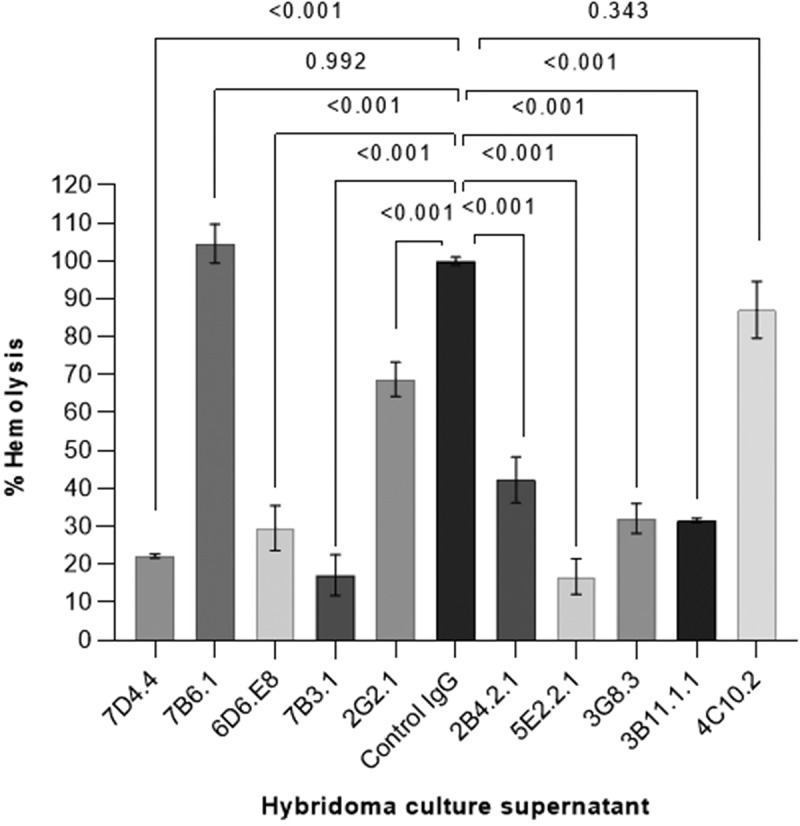
Hemolysis assay on sheep erythrocytes co-incubated with culture supernatants from selected anti-Cdlys-producing hybridoma cell lines and Candidalysin (20 μM). The % hemolysis is calculated as: (abs 540 nm sample/abs 540 nm control IgG hybridoma supernatant) ×100. Data are presented as mean ± SEM from three independent experiments (*n* = 3). Error bars represent the standard error of the mean. *p* values were calculated using one-way ANOVA with Dunnett’s multiple-comparaison test.

Candidalysin exerts a strong cytolytic effect on epithelial cells [8]. *In vitro*, it has also been shown to demonstrate hemolytic activity at micromolar concentrations [30,31]. To select anti-Cdlys-neutralizing mAbs, we studied their ability to block the hemolytic activity of the toxin. To do this, red blood cells from sheep were suspended in the culture supernatant of each selected hybridoma cell line supplemented with 20 μM of Candidalysin. The level of hemoglobin released was measured using spectrophotometry (λ = 540 nm) and compared to that of a negative control, including the supernatant of the hybridoma cell line producing the anti-Hyr1 4F3.2.1 mAb. Of the ten tested supernatants, eight significantly decreased hemolysis, suggesting that the mAbs they contained were efficient to neutralize the toxin and reduce its hemolytic activity (Figure 4). Among the most efficient, anti-Cdlys mAbs produced by 7D4.4, 7B3.1, 5E2.2.1, and 3B11.1.1 hybridoma cell lines were arbitrarily selected for further analysis.

### Characterization of isotype and binding affinity of selected anti-Hyr1 and anti-cdlys mAbs

**Figure 5.**
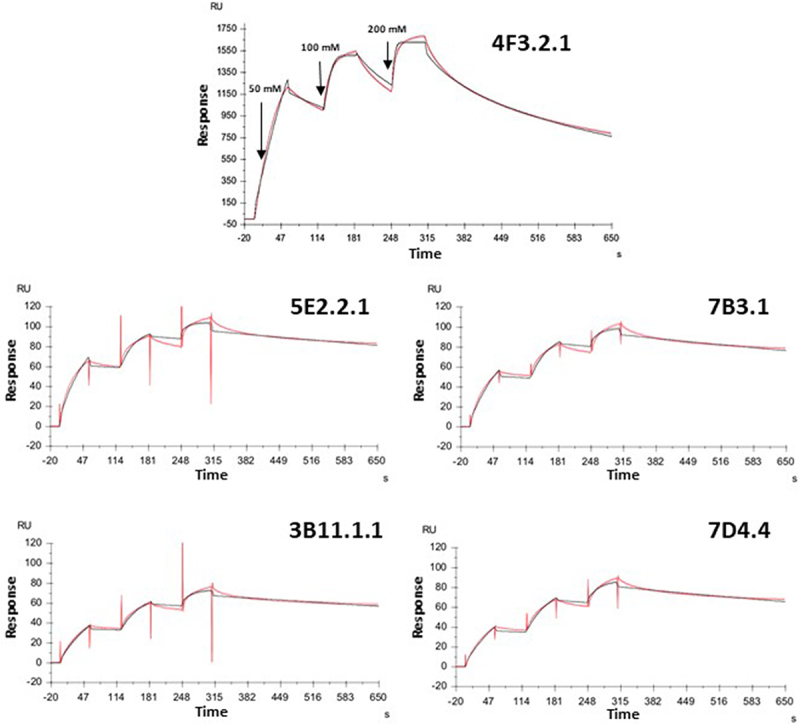
Sensorgrams of surface plasmon resonance (SPR) measurements for the determination of anti-Hyr1 4F3.2.1 mAb’s binding affinity to Hyr1_(aa 274–322)_ and anti-Cdlys 3B11.1.1, 5E2.2.1, 7B3.1, and 7D4.4 mAbs’ binding affinity to Candidalysin. For each measurement, the antigen was immobilized on a gold biosensor surface (Biacore sensor chip, CM5). Each mAb was then injected at increasing concentrations of 50 nM, 100 nM, and 150 nM for 60 s, followed by buffer injection for 240 s. To determine kinetic constants, the data were fitted by a 1:1 Langmuir interaction model using the sofware bioevaluation. Fit curves are shown in red.

All selected mAbs were purified from the cell culture supernatant by protein G affinity chromatography. Their isotypes were determined, and Surface Plasmon Resonance (SPR) was used to measure their equilibrium dissociation constant (K_D_) for the respective antigenic target. For SPR experiments, sensor chips were coated with the synthetized Hyr1_(aa 274–322)_ (for anti-Hyr1 4F3.2.1 analysis) or Candidalysin peptides (for anti-Cdlys 3B11.1.1, 5E2.2.1, 7B3.1 and 7D4.4 mAbs analysis) by amine coupling, and probed with sequentially increasing concentrations of purified mAbs (50, 100, 200 nM). The binding kinetics are shown on the sensograms (Figure 5); acquired data were analyzed using the Langmuir 1:1 model of interaction. Results revealed that anti-Hyr1 4F3.2.1 is an IgG2a isotype displaying a high-affinity for Hyr1_(aa 274–322)_ with a K_D_ of 2.8x10^−9^ M. All anti-Cdlys mAbs were IgG1 isotypes with very close affinity for the Candidalysin peptide ranging from 3.1x10^−9^ M to 1.4x10^−9^ M. The anti-Cdlys 5E2.2.1 displaying the highest affinity was selected for further studies (Table 3).

**Table 3.** Isotype and binding affinity of the selected anti-Hyr1 4F3.2.1 mAb and anti-Cdlys 3B11.1.1, 5E2.2.1, 7B3.1, and 7D4.4 mAbs.

			Affinity binding		
mAb	Target antigen	Isotype	ka (M^−1^ s^−1^)	kd (s^−1^)	K_D_ (M^−1^)
4F3.2.1	Hyr1_(aa 274–322)_	IgG2a	2.1 × 10^6^	5.8 × 10^−3^	**2.8 ×10**^**−9**^
3B11.1.1	Cdlys	IgG1	2.3 × 10^5^	5.0 × 10^−4^	**2.2 ×10**^**−9**^
5E2.2.1	Cdlys	IgG1	3.4 × 10^5^	4.7 × 10^−4^	**1.4 ×10**^**−9**^
7B3.1	Cdlys	IgG1	2.6 × 10^5^	5.5 × 10^−4^	**2.1 ×10**^**−9**^
7D4.4	Cdlys	IgG1	1.9 × 10^5^	6.0 × 10^−4^	**3.1 ×10**^**−9**^

### In vitro evaluation of the functional activity of anti-Hyr1 4F3.2.1 and anti-cdlys 5E2.2.1 mAbs

#### Opsonophagocytic activity of anti-Hyr1 4F3.2.1

The opsonizing capacity of anti-Hyr1 4F3.2.1 was evaluated by a phagocytosis test using an *in vitro* cellular model previously developed to study the cellular interactions between *Candida* yeasts and phagocytes [32].

**Figure 6.**
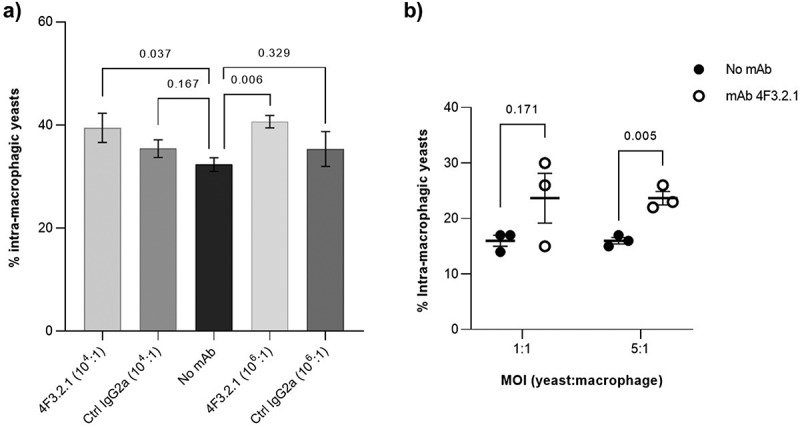
Evaluation of the opsonophagocytic activity of anti-Hyr1 4F3.2.1 mAb. (a) J774 macrophage cells were infected for 1 h with CFW-labelled blastospore *C. albicans* cells (MOI 1:1, yeast to macrophage) in a filamentation-inducing medium (RPMI/FBS 10%) either in the absence or in the presence of anti-Hyr1 4F3.2.1 mAb or a mAb IgG2a isotype control mAb using different ratios of mAb:yeast. (b) J774 macrophage cells were infected for 1 h with CFW-labelled *C. albicans* hyphal cells with different MOI (1:1 or 5:1, yeast to macrophage) in a filamentation-inducing medium (RPMI/FBS 10%) either in the absence or in the presence of anti-Hyr1 4F3.2.1 mAb (ratio 10^6^:1, mAb to fungal cell). The percentage of phagocytosis is determined as the ratio of the fluorescence emitted by internalized fungal cells (after quenching the fluorescence of externalized cells with trypan blue) to the initial total fluorescence ×100. Data are presented as mean ± SEM from three independent experiments (*n* = 3). Error bars represent the standard error of the mean. *p* values were calculated using a Student’s t-test.

Murine macrophage J774 cells were infected with Calcofluor White (CFW)-labeled yeast cells (multiplicity of infection (MOI) 1:1, yeast to macrophage) in a filamentation-inducing medium (RPMI/FBS 10%) and incubated with anti-Hyr1 4F3.2.1 or with an isotypic control mAb. Arbitrarily, two ratios for mAbs to yeasts of 10^4^:1 and 10^6^:1 were tested. Following infection, trypan blue was added to quench the fluorescence of the unphagocytosed CFW-labeled yeasts specifically to detect the fluorescence of the internalized yeasts. As expected, no increase in intramacrophagic yeasts was observed when cells were treated with the isotypic control mAb. By contrast, the incubation of yeasts with anti-Hyr1 4F3.2.1 resulted in an increase in the number of phagocytosed yeasts by 7 and 8%, depending on the antibody-to-cell ratio tested (Figure 6(a)). These results demonstrated that anti-Hyr1 4F3.2.1 effectively elicits antibody-dependant cellular phagocytosis (ADCP) following the opsonization of *C. albicans* cells.

A potential issue of this assay is that some yeasts may have been phagocytosed in their blastospore form, and not exclusively as opsonized hyphae. To directly assess whether anti-Hyr1 4F3.2.1 possessed opsonic properties, we conducted a new experiment in which yeasts were grown under conditions that induced filamentation (RPMI/FBS 10%, 37°C) for 30 minutes (previously demonstrated as the earliest stage of hyphae recognized by the mAb 4F3.2.1 (Figure 2(b)) before macrophage infection. Two MOI were used in this experiment. The results (Figure 6) show that exposing macrophages to an excess of yeasts (MOI 5:1, yeasts to macrophages) treated with anti-Hyr1 4F3.2.1 leads to a significant increase in the percentage of internalized hyphae (7%). A similar increase was observed for the MOI 1:1, but it was not statistically significant (Figure 6(b)). Overall, our findings demonstrate the opsonophagocytic activity of anti-Hyr1 4F3.2.1 mAb which effectively and selectively enhances the phagocytosis of *C. albicans* hyphae *in vitro* by murine J774 macrophages.

#### Neutralizing activity of anti-cdlys 5E2.2.1

As previously reported [8], Candidalysin exhibits cytolytic activity during mucosal infections, causing damage to epithelial cells and triggering host signaling pathway activation leading to the production of pro-inflammatory cytokines, chemokines, and antimicrobial peptides. During infections, Candidalysin is secreted within the invasion pocket formed by the invagination of the hyphae into epithelial cells. Once accumulated, the peptide toxin generates cellular damage by forming pores through cell membranes leading to an influx of calcium and to the release of intracytoplasmic components into the extracellular environment, such as lactate dehydrogenase (LDH) [8,33].

**Figure 7.**
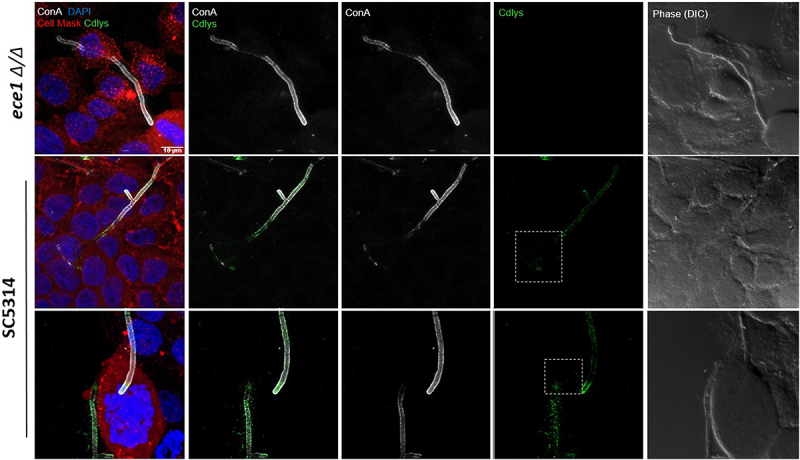
Immunofluorescence imaging of anti-Cdlys 5E2.2.1 mAb. Monolayer A431 vaginal epithelial cells were infected with *C. albicans* SC5314 (WT) (two last panels) or *ece1*∆/∆ mutant strain (first panel) and incubated for 180 min under filamentation-inducing conditions. The plasma membrane of A431 cells was stained with CellMask^TM^ marker (red). Following fixation, extracellular, noninvasive fungal components were stained with concanavalin a (ConA, white) and Candidalysin was detected with anti-Cdlys 5E2.2.1 mAb (detected with a green fluorescent secondary antibody). Dashed boxes highlight areas of hyphal invagination within epithelial cells, indicating localized Candidalysin secretion at these invasion sites.

**Figure 8.**
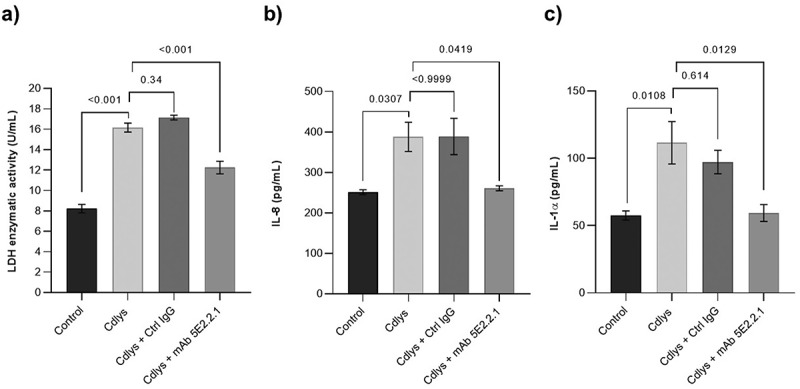
Evaluation of the neutralizing activity of anti-Cdlys 5E2.2.1 mAb on the cytolytic and pro-inflammatory effects of Candidalysin. A431 vaginal epithelial cells were exposed to Candidalysin for 24 h, either alone or in the presence of anti-Cdlys 5E2.2.1 mAb, or in the presence of an IgG isotype control mAb. (a) Cell damage was quantified by measuring the enzymatic activity of LDH released into the culture supernatant. Quantification of IL-8 (b) and IL-1α (c) cytokines secreted by A431 epithelial cells in response to Candidalysin. Data are presented as mean ± SEM from three independent experiments (*n* = 3). Error bars represent the standard error of the mean. *p* values were calculated using one-way ANOVA with Dunnett’s multiple-comparaison test. For each test, the first control condition corresponds to A431 cells not exposed to Candidalysin.

To further characterize the anti-Cdlys 5E2.2.1 mAb, we initially confirmed its ability to bind to Candidalysin released by hyphae within human A431 epithelial cell invasion pockets by immunofluorescence assays *in cellulo* (Figure 7). We then investigated the neutralizing properties of anti-Cdlys 5E2.2.1 on Candidalysin-induced cytotoxic effects. For this purpose, A431 epithelial cells were exposed for 24 h to Candidalysin alone or to Candidalysin treated with either anti-Cdlys 5E2.2.1 or a mAb control. We then measured the enzymatic activity of LDH and the concentration of the realeased pro-inflammatory cytokines IL-1α and IL-8. As expected, LDH activity significantly decreased when cells were co-incubated with Candidalysin and anti-Cdlys 5E2.2.1, compared to cells incubated with the toxin alone or with the toxin and the control mAb (Figure 8(a)). Moreover, while Candidalysin alone induces a significant release of pro-inflammatory cytokines, no significant difference in IL-8 and IL-1α concentrations was observed in the presence of anti-Cdlys 5E2.2.1 compared to the control condition (Figure 8(b, c)). Overall, these results demonstrated the neutralizing activity of the anti-Cdlys 5E2.2.1 mAb against the Candidalysin toxin through the inhibition of both the cytolytic and the pro-inflammatory effects of the toxin *in cellulo*.

### In vivo evaluation of the prophylactic efficacy of anti-cdlys 5E2.2.1 and anti-Hyr1 4F3.2.1 for preventing murine vaginal candidiasis

#### Development and characterization of *C.* *albicans* bioluminescent strain

**Figure 9.**
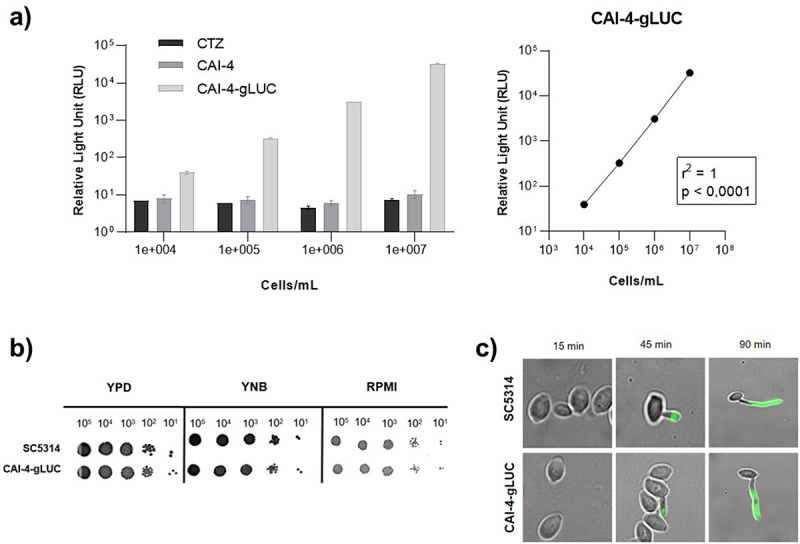
Bioluminescent and phenotypic properties of CAI-4-gLUC strain. (a) Graphical representation and linear regression of bioluminescence emission as a function of cell concentration for CAI-4-gLUC. (b) Effect of *Gaussia princeps* luciferase expression on CAI-4-gLUC growth using drop test method on YPD, YNB and RPMI media. (c) Immunofluorescence labeling of anti-Hyr1 4F3.2.1 mAb in SC5314 (WT) and CAI-4-gLUC *C. albicans* strains during elongation.

To conduct real-time monitoring of the progression of infection in a murine model of vaginal candidiasis, we generated the *C. albicans* bioluminescent strain CAI-4-gLUC by integrating the *C. albicans*-optimized *Gaussia Luciferase* CIp10:Act1p-gLUC59 reporter gene into the *RPS1* locus of the CAI-4 strain, as previously described [27]. To assess the number of gLUC-expressing cells required to obtain a detectable luminescent signal, a fixed concentration of coelenterazine (CTZ) was added to both empty wells and to ten-fold dilution series of CAI-4-gLUC cells. The signal intensity measured from 10^4^ or more CAI-4-gLUC cells was found to be significantly higher than the background signal observed from auto-oxidation of CTZ alone or incubated with non-luminescent CAI-4 cells (Figure 9(a)). Additionally, we showed that the intensity of the luminescent signal was fully proportional to the number of cells, as evidenced by a linear correlation coefficient of R^2^ = 1 and a p-value of less than 0.0001.

Before conducting animal experiments using the CAI-4-gLUC strain, we also examined whether luciferase expression could impact cellular growth. A drop test performed with the CAI-4-gLUC and the WT strain (SC5314) revealed no changes in cellular growth on complete YPD medium, minimal YNB medium, and RPMI medium (Figure 9b).

Because luciferase expressed from the CIp10:Act1p-gLUC59 reporter is localized at the cell surface of *C. albicans*, we considered that its expression could induce steric hindrance within the cell wall and potentially affect the exposure of other cell wall proteins, thus interfering with the binding of anti-Hyr1 4F3.2.1. To address this question, anti-Hyr1 4F3.2.1 binding was monitored during the elongation of the CAI-4-gLUC cells (15, 45, 90 min) in filamentation induction medium (RPMI/FBS 10%, 37°C). As shown in Figure 9(c), no difference in the binding kinetics of 4F3.2.1 between the bioluminescent strain CAI-4-gLUC and the wild-type strain SC5314 could be observed, suggesting that the expression of luciferase at the cell wall did not interfere with the binding capacity of the mAb.

### Study of the prophylactic therapeutic efficacy of anti-cdlys 5E2.2.1 and anti-Hyr1 4F3.2.1 in vivo

**Figure 10.**
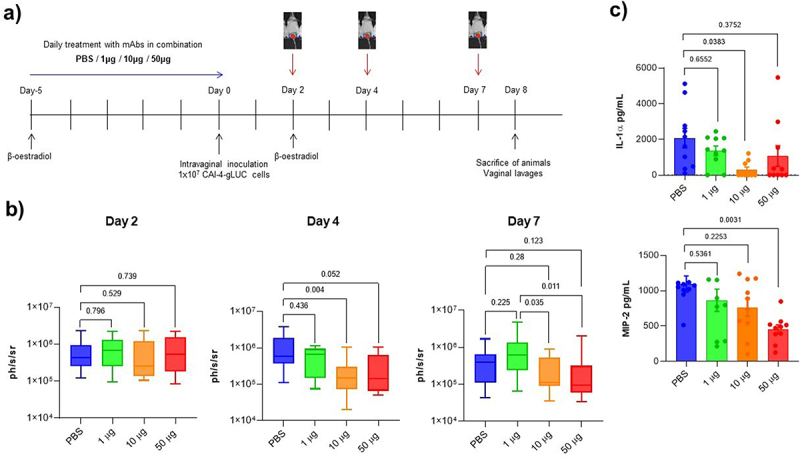
Evaluation of the efficacy of passive immunization prophylaxis using a combination of anti-Cdlys 5E2.2.1 and anti-Hyr1 4F3.2.1 mAbs at different concentrations in a mouse model of VVC. (a) Timeline of the vaginal infection model. (b) Quantification of bioluminescence from CAI-4-gLUC cells on days 2, 4, and 7 following completion of mAb prophylaxis treatment (*n* = 10 animals per group) or PBS control (*n* = 10 animals). Error bars represent the standard error of the mean. *p* values were calculated using mann-whitney U test. (c) Quantification of IL-1α and MIP-2 immunomodulators in vaginal lavage samples from treated mice. Error bars represent the standard error of the mean. *p* values were calculated using one-way ANOVA with Tukey’s multiple-comparaison test.

The prophylactic therapeutic efficacy of anti-Cdlys 5E2.2.1 and anti-Hyr1 4F3.2.1 was studied in an experimental mouse model of vaginal candidiasis according to the timeline shown in Figure 10(a). Mice (ten animals per group) in a pseudo-estrus condition were intravaginally treated for six consecutive days, either with the mAbs in combination (1, 10 or 50 μg of each mAb) or with PBS (negative control). To visualize the fungal infection in living animals, mice were intravaginally challenged with 1 × 10^7^ cells of CAI-4-gLUC strain, and the luciferase signal was monitored over time following local administration of CTZ using a PhotonIMAGER RT imager (Biospace) until the seventh day following infection. As shown in Figure 10(b), fungal load was significantly reduced in infected animals at day four after treatment with 10 μg of each mAb, but not with 1 μg or 50 μg, compared to animals treated with PBS. This trend was maintained at day seven post-infection, although the reduction was no longer statistically significant compared to the PBS control group. However, a reduction in fungal load between mice groups treated with 10 or 50 μg on the one hand and animals treated with 1 μg on the other hand was still observed seven days after stopping the treatment.

Eight days post-infection, the animals were sacrified and vaginal washes were performed to quantify the pro-inflammatory cytokine IL-1α and the chemokine MIP-2 (functional equivalent in mouse of human IL-8) whose expression were shown to be strongly induced in response to Candidalysin. The results presented in Figure 10c show that the concentration of IL-1α was significantly lower in the group treated with 10 μg of each mAb than in the control group. This significant reduction was not observed in the group treated with 50 μg, which may be explained by a greater variability in mesurement points in this experimental group, although the mean was still lower than in the PBS-treated control group. The decrease of IL-1α production indicates a reduction of the inflammatory state of the vaginal mucosa, which could be the result of the neutralization of Candidalysin by anti-Cdlys 5E2.2.1. The concentration of chemokine MIP-2 was significantly reduced in animals treated with 50 μg of each mAb. This may be the consequence of both the neutralization of Candidalysin and the decrease in the recruitment of phagocytic cells into the vaginal lumen. Overall, these results demonstrate that the preventive treatment of the vaginal epithelium with a combination of anti-Cdlys 5E2.2.1 and anti-Hyr1 4F3.2.1 mAbs reduces colonization by fungal cells and decreases inflammation in the vaginal environment during an episode of induced VVC in mice.

## Discussion

Infectious diseases caused by *Candida* yeasts represent a serious global health challenge due to the emergence of strains and species resistant to conventional antifungal therapies. This issue is particularly critical in the case of vaginal candidiasis, especially its recurrent form, which requires repeated antifungal treatments while not guaranteeing long-term eradication of infectious episodes. Against this backdrop, it was clearly of interest to explore alternative therapeutic strategies in treating vaginal candidiasis. For a number of years, immunotherapy through vaccination has been considered as a promising means to treat and prevent *Candida* infections [34]. Although no anti-*Candida* vaccines are currently available on the market, two recombinant protein-based vaccines have progressed to Phase 1 clinical trials. The first candidate vaccine, NDV-3A, consists of a recombinant version of the N-terminal domain of the Als3p protein. Preclinical studies have shown its preventive efficacy against superficial and systemic infections caused by *C. albicans* [35]. Phase 1b/2a clinical trials focusing on RVVC have further shown that intramuscular administration with NDV-3A significantly reduces the frequency of infectious episodes in women under the age of 40 years [36]. This protective effect correlates with a strong activation of adaptive immune responses, mediated by both cellular and humoral pathways. The second vaccine, named PEV7, contained a recombinant version of Sap2 protein in a virosomal formulation and has shown itself to be effective in eliciting the production of memory B cells in a rat model of *Candida* vaginitis when administred via the intravaginal route [37].

However, since the recent discovery of the peptide fungal toxin Candidalysin, a number of scientific studies have converged on the idea that VVC episodes may in fact result from an immunopathological disorder characterized by an exacerbated inflammatory response in the host [8]. The establishment of an inflammatory loop within the vaginal lumen is believed to result from both the activation of the inflammatory process, primilarily driven by Candidalysin, and from a local dysfunction of innate immunity, where the recruited PMNs are unable to efficiently recognize *C. albicans* due to neutrophil anergy [10].

The aim of this study was to test passive immunization as an alternative immunotherapy for enhancing resistance to vaginal *C. albicans* infections by counteracting the uncontrolled inflammation resulting from impaired neutrophil recognition during (R)VVC. Recent studies identified the parietal Hyr1 protein as a promising immunotherapeutic target. Hyr1 is specifically expressed by the hyphal pathogenic form of *C. albicans* and has been described as a critical factor in the ability of *Candida* to evade host defenses mediated by phagocytes [22,24]. Furthermore, passive immunization using anti-Hyr1 mAbs derived from B cell cultures from patients with *C. albicans* infections has shown promising results in stimulating macrophage opsonophagocytic activity in a murine model of disseminated candidiasis [38]. However, although passive immunization has been shown to be effective in treating systemic fungal infections [24,38–41], its efficacy against superficial fungal infections remains underexplored. This is primarily due to the fact that antibodies administered via the conventional systemic route are not secreted at mucosal surfaces [42]. Nevertheless, an increasing number of studies have demonstrated the applicability of developing innovative local antibody delivery systems, such as mucoadhesive tablets [43], films [44], nebulizers [45], and semi-solid gel formulations [46], that could offer promising opportunities to optimize the delivery of antibodies at mucosal surfaces.

To set up the passive mucosal immunization strategy for the prevention of RVVC, we hypothesized that simultaneously targeting Candidalysin and the Hyr1 protein could elicit a synergistic effect on the host’s immune response. Specifically, this dual targeting approach is expected to modulate the intensity of the inflammatory response while restoring the phagocytic efficacy of vaginal innate immune cells.

We employed traditional hybridoma technology for the production and the screening of murine mAbs targeting the N-terminal region of Hyr1 and the fungal toxin Candidalysin. We first selected the anti-Hyr1 4F3.2.1 mAb. Through immunofluorescence experiments, we confirmed that this mAb is highly specific to the hyphal form of *C. albicans*. It binds to the recombinant Hyr1_(274–322)_ antigen with a K_D_ value of 2.8 nM. However, cross-binding to other natural antigens expressed on the surface of *C. albicans* filaments has also been observed. Hyr1 is part of a group of “hyphally-regulated” proteins that belong to a larger family (IFF family for “Individual protein file Family File”), which includes 12 proteins [47]. The fact that anti-Hyr1 4F3.2.1 can recognize other *C. albicans* cell wall proteins is ultimately not surprising. The peptide used in this work for immunization is derived from the N-terminal region of the Hyr1 protein (aa 274–322), known to be highly conserved among all IFF family members, especially for the first 350 amino acids [48]. Moreover, this region is located at the base of a ß-helical extracellular structure of Hyr1, which is encountered in many surface proteins of lectin-like or, more generally, adhesin-like nature, expressed in various microorganisms such as viruses, bacteria, and fungi [23]. However, it is not problematic that the selected antibody recognizes other cell wall epitopes, so long as they are specific to the hyphal form of *C. albicans*.

The anti-Hyr1 4F3.2.1 mAb is an IgG2a isotype. This isotype is an important parameter to consider when the therapeutic strategy relies on the activation of antibody-dependent cellular functions such as ADCC (antibody-dependent cellular cytotoxicity), CDC (complement-dependent cytotoxicity), or ADCP (antibody-dependent cellular phagocytosis). In mice, IgG2 is desirable since the specific murine FcγRIV receptor expressed by monocyte/macrophages and neutrophils has a high exclusive binding affinity for IgG2a/b. In mice, this receptor is primilarly involved in the ADCC activation process [49]. Mouse IgG2a also strongly binds to murine FcγRI expressed on monocytes/macrophages and dendritic cells, whose main function is to activate phagocytosis via ADCP.

Anti-Hyr1 4F3.2.1 was developed to bypass the anti-phagocytic property of Hyr1 [22]^(p1)^. To study the opsonizing capacity of the mAb, we used a phagocytosis assay that involves murine macrophages (J774) and calcofluor-white labeled yeasts at the early stage of their filamentation [32]. This assay allowed us to confirm that opsonization of hyphae by anti-Hyr1 4F3.2.1 enhanced their phagocytosis by macrophages *in vitro*.

The second selected mAb is an IgG1 mAb referred to as anti-Cdlys 5E2.2.1, which is specifically directed against the peptide toxin Candidalysin. This mAb has a high binding affinity to Candidalysin with a K_D_ value of 1.4 nM. As previously described, due to its nature as a pore-forming toxin, Candidalysin compromises the permeability of the epithelial cell membrane [8,33,50]. The resulting cellular damage activates cellular signaling pathways and triggers the secretion of immunomodulators and the recruitment of immune cells. Given the critical role of this toxin during VVC, our initial focus was to assess the neutralizing activity of anti-Cdlys 5E2.2.1 on cell integrity and mucosal immune activation. We showed that anti-Cdlys 5E2.2.1 had a robust neutralizing effect on the lytic activity of the toxin. This was initially observed in red blood cells from sheep and then confirmed in human A431 vulvar cells, where a significant decrease in the cellular damage marker LDH level was observed during co-incubation of the toxin with this mAb. The neutralizing activity of anti-Cdlys 5E2.2.1 against the pro-inflammatory property of the toxin was shown *in vitro* on A431 cells by its capacity to significantly decrease the release of cytokines IL-1α and IL-8 following the treatment of cells with Candidalysin.

Lastly, we evaluated the efficacy of passive immunization using the combination of anti-Hyr1 4F3.2.1 and anti-Cdlys 5E2.2.1 for the prevention of experimental VVC in mice. The use of a bioluminescent strain of *C. albicans* (CAI-4-gLUC) that expressed *Gaussia princeps* luciferase enabled us to multiply mesurements over time in anesthetized animals [27]. We observed that prophylactic treatment by intravaginal administration of both mAbs in combination reduced the fungal burden as well as the levels of IL-1α cytokine and MIP-2 chemokine during the first week post-infection. This result constitutes the first evidence that prophylactic treatment with anti-Hyr1 4F3.2.1 and anti-Cdlys 5E2.2.1 mAbs attenuates *C. albicans* vaginal infections. However, a key question is the relative contribution of each antibody to the overall attenuation of VVC in mice. The observed reduction in pro-inflammatory cytokines may result from Candidalysin neutralization, decreased fungal burden via opsonization, enhanced neutrophil activity, or a combination of these mechanisms. Further insight could be gained by quantifying innate immune cell populations and neutrophil effector functions (ROS, NADPH oxidase, proteases, neutrophil extracellular traps) to determine whether the treatment promotes reversal of neutrophil anergy. Neutropenic mice could serve as negative controls in this context. However, mice are not natural hosts for *Candida*, and VVC in this model requires estradiol treatment. Moreover, the vaginal microbiota of mice is not dominated by *Lactobacillus* species as in women, and consequently, the vaginal pH is neutral, whereas it is acidic in women (pH 4.5). These differences limit the extent to which passive prophylactic immunization efficacy in mice can be extrapolated to humans. Future work should consider alternative animal models and humanization of the selected antibodies for *in vitro* characterization under acidic conditions (biological activity, binding affinity, stability) that better mimic the human vaginal environment. Globally, our study provides promising new data that supports further clinical development of these mAbs for the preventive treatment of VVC, particularly with regard to patients who suffer from recurrent episodes of VVC.

## ARRIVE guidelines statement

The authors confirm that they adhere to the ARRIVE guidelines (https://doi.org/10.17632/3r9dghk85h.2).

## Data Availability

The data supporting the findings of this study are available at Mendeley Data (https://doi.org/10.17632/3r9dghk85h.2).
