# Correction

**DOI:** 10.1080/21505594.2025.2580806

**Published:** 2025-11-04

**Article title**: Mucosal passive immunization with monoclonal antibodies targeting Candidalysin and Hyr proteins attenuates vaginal candidiasis in mice

**Authors**: Julie Auffray, Nicolas Biteau, Anne Cayrel, Denis Dacheux, Hassana Hsein, Pierre Tchoreloff & Thierry Noel

**Journal**: *Virulence*

**DOI**: http://dx.doi.org/ 10.1080/21505594.2025.2569629

When the article was originally published, values that should have been formatted as superscripts were mistakenly presented as normal values within brackets. This has now been corrected, and the updated article has been republished online.

